# Risks and rewards of using prepaid vs. postpaid incentive checks on a survey of physicians

**DOI:** 10.1186/s12874-018-0565-z

**Published:** 2018-10-11

**Authors:** Kristine Wiant, Emily Geisen, Darryl Creel, Gordon Willis, Andrew Freedman, Janet de Moor, Carrie Klabunde

**Affiliations:** 1 RTI International, 3040 East Cornwallis Road, Research Triangle Park, NC 27709 USA; 2 RTI International, 6110 Executive Boulevard, Suite 902, Rockville, MD 20852 USA; 3 National Cancer Institute, 9609 Medical Center Drive, Rm 3E228, MSC 9762, Bethesda, MD 20892-9762 USA; 4 National Cancer Institute, 9609 Medical Center Drive, Room 4E226, Rockville, MD 20850 USA; 5 National Cancer Institute, 9609 Medical Center Drive, Room 3E438, Bethesda, MD 20892-9764 USA; 6 National Institutes of Health, 6100 Executive Boulevard, Suite 2B03, Rockville, MD 20852 USA

**Keywords:** Physicians, Surveys, Survey incentives, Response rates, Precision medicine, Oncologists, Provider surveys

## Abstract

**Background:**

Survey researchers use monetary incentives as a strategy to motivate physicians’ survey participation. Experiments from general population surveys demonstrate that prepaid incentives increase response rates and lower survey administration costs relative to postpaid incentives. Experiments comparing these two incentive strategies have rarely been attempted with physician samples.

**Methods:**

A nationally representative sample of oncologists was recruited to participate in the National Survey of Precision Medicine in Cancer Treatment. To determine the optimal strategy for survey incentives, sample members were randomly assigned to receive a $50 prepaid incentive check or a $50 promised (postpaid) incentive check. Outcome measures for this incentives experiment include cooperation rates, speed of response, check-cashing behavior, and comparison of hypothetical costs for different incentive strategies.

**Results:**

Cooperation rates were considerably higher for sample members in the prepaid condition (41%) than in the postpaid condition (29%). Similar differences in cooperation rates were seen for physicians when stratified by region, size of the physician’s metropolitan statistical area, specialty, and gender by age. Survey responders in the prepaid condition responded earlier in the field period than those in the postpaid condition, thus requiring fewer contacts. In the prepaid group, 84% of sample members who responded with a completed survey cashed the incentive check and only 6% of nonresponders cashed the check. In the postpaid condition, 72% of survey responders cashed the check; nonresponders were not given a check. The relatively higher cooperation rates and earlier response of the responders in the prepaid condition was associated with a 30% cost savings for the prepaid condition compared to the postpaid incentive condition.

**Conclusions:**

The results of this study suggest that the rewards of offering physicians a prepaid incentive check outweigh the possible risks of nonresponders cashing the check. The relative cost benefit of this strategy is likely to vary depending on the amount of the incentive relative to the costs of additional contact attempts to nonresponders.

## Background

Survey data from physicians are an important source of information on physician practices and opinions in health care delivery. Because these data are often not available from any other source, surveys of physicians have become increasingly common in recent decades. However, participation in physicians’ surveys has been declining [1–4]. Thus, finding ways to bolster participation is a key concern amongst those who survey physicians.

Surveying physicians can be particularly challenging because physicians are busy and are surrounded by “gatekeepers” (office managers, receptionists, nurses, etc.) who may limit an interviewer’s access to scheduling an appointment to conduct an interviewer-administered survey [5]. For this reason and budget constraints, most physician surveys are self-administered, relying on contact via mail, e-mail, or both. Survey researchers must find a way to motivate survey participation without direct verbal interaction.

Monetary incentives are one of a host of tools that survey researchers employ to motivate survey participation, and the use of incentives is recommended for physician surveys [5–8]. Incentives are effective in increasing response rates to surveys in all modes and have been found to be particularly impactful when used on self-administered surveys and when prepaid [9]. These effects are often explained with concepts stemming from social exchange theory. For example, Dillman, Smyth, and Christian [10] posit that prepaid incentives create a feeling of goodwill and encourage recipients to reciprocate the gesture by completing the survey. The authors note that the novel gesture of a prepaid incentive can also encourage recipients to read and consider the researchers’ request, rather than simply throwing the package away.

Experiments conducted within surveys of the general population have demonstrated that prepaid incentives can increase response rates and decrease the number of survey prompts compared with postpaid incentives [9]. Yet with physician surveys, incentives are typically larger and response rates are generally lower relative to surveys of the general population. Only two studies with physicians have compared prepaid versus postpaid incentives. Although both Delnevo et al. [11] and James et al. [12] found that prepaid incentives bolstered response rates, these studies both leave room for additional investigation of the cost implications of incentive timing.

A potential risk of prepaid incentives is the possibility of sample members keeping the incentive without returning a completed survey. For example, Delnevo et al. [11] found that costs were higher for prepaid condition because of the sunk costs in providing prepaid gift cards to physicians who did not ultimately respond. However, others have employed strategies to reduce this risk. Chen et al. [13] used a strategy that allowed the investigators to reclaim funds from prepaid gift cards that were not redeemed by the physicians. They found that 63.5% of the responding clinicians redeemed the gift card and only one nonresponding clinician redeemed the card. A similar strategy for prepaid incentives entails providing this in the form of a personalized check so cost is incurred only if the sample member cashes the check. When using prepaid checks, Hogan [14] found that only 44% of the physicians in the sample cashed the prepaid check. Of those who cashed the check, 89.5% were survey responders. Moreover, 35.2% of survey responders did not cash the check. In the Hogan study, all sample members were prepaid, so it is unclear how this might compare to the check-cashing behavior of postpaid respondents.

The literature supports the use of prepaid incentives for improving response rates amongst physician samples. However, few studies have made *experimental* comparisons between pre- and postpaid incentive strategies in physician surveys, and questions remain regarding whether prepaid incentives are also cost effective. In the present paper, we use experimental design to address the following questions:

1. What is the effect of incentive timing on survey participation, and does that differ by physician characteristic?
2. What are the financial risks of sending a prepaid check, and does that risk differ by physician characteristic?

## Methods

Data for this study come from the National Survey of Precision Medicine in Cancer Treatment, sponsored by the National Cancer Institute of the U.S. Department of Health and Human Services. Conducted by RTI International between February and June of 2017, this was the first nationally representative survey of oncologists about their experiences, attitudes, and recommendations concerning genomic testing in oncology. The National Survey of Precision Medicine in Cancer Treatment was fielded among a nationally representative sample of medical oncologists—including hematologists, hematologists/oncologists, and oncologists drawn from the American Medical Association Physician Masterfile.

We randomly assigned 75% of the sample to receive a prepaid $50 incentive check and the remaining 25% to receive a $50 promised (postpaid) incentive check. Given a threshold of power 0.8, we determined the 75% prepaid and 25% postpaid allocation would meet the analytic requirements of detecting a difference of 4 percentage points or more. Due to differences in eligibility rates, this resulted in 72% of the eligible sample being in the prepaid group and 28% in the postpaid group.

To be eligible for the survey, physicians must have treated cancer patients within the previous 12 months. Trained telephone interviewers conducted screening calls with offices of sampled physicians to verify eligibility for survey participation and contact information. The protocol for conducting the screening calls was identical for physicians in the prepaid and postpaid incentive conditions.

Upon completion of screening telephone calls, the survey was fielded among the 3465 eligible physicians. The first survey mailing included a personalized cover letter, an endorsement letter from the American Society of Clinical Oncology, a pen with the study’s name printed on it, a 12-page questionnaire booklet, and a business reply envelope. The first mailing to those in the prepaid condition included a $50 prepaid honorarium check; the letter in the mailing to those in the postpaid condition notified the sample members that we would mail them a $50 honorarium check after we received their completed response.

After the first mailing, nonresponders received additional prompts to complete the survey. When e-mail addresses were available, we sent up to two e-mail invitations with a personalized link to a web version of the survey. We also sent up to two additional survey packets, each containing a reminder letter, a replacement questionnaire, and a business reply envelope. Finally, we followed up with a reminder phone call to nonresponders. For respondents in the postpaid condition, the e-mail invitations, additional mailings, and reminder call script all stated that we would send a $50 honorarium check upon receipt of the physician’s completed questionnaire. For respondents in the prepaid condition, only the first e-mail invitation and the reminder call script explicitly reminded the sample members that the initial mailing they received included a $50 honorarium check. See Geisen et al. [15] for additional details about the sampling and data collection approach.

The National Survey of Precision Medicine survey achieved an overall response rate of 29.4%, using the American Association for Public Opinion Research’s (AAPOR’s) response rate 3 [16], 62, p., which incorporates estimates of the proportion of eligible cases in the unknown eligibility cases. We estimated the proportion of eligible cases based on the observed eligible proportion from the cases with known eligibility.

Response rate 3 is the product of contact rate 2 [16], 65, p. and cooperation rate 1 [16], 63, p. “Contact rate” for the National Survey of Precision Medicine in Cancer Treatment refers to the proportion of the sample that we could successfully contact during the screening call; cooperation rate refers to the outcome of the subsequent survey invitations that we sent to physicians who were identified as eligible during the screening call. Because the protocol for screening calls was identical for physicians in the pre- and postpaid conditions, contact rates were not affected by timing of the incentive payments. Only the survey invitations and prompts varied by timing of incentive. We therefore focus on cooperation rate in this study rather than contact rate or response rate.

To assess and minimize potential bias, we used information available from the sampling frame. Using available variables (primary specialty, metropolitan statistical area category, gender/age combination, and Census region) believed to be associated with the outcomes of interest, we implemented two adjustments to the design weight. The first adjustment was a noncontact adjustment. The second adjustment was a noncooperation adjustment and created the final analysis weight. Throughout the weight adjustment process, we monitored the unequal weighting effect and effective sample size. In addition, we calculated the R-indicator [17] overall, for incentive group, and for the stratification variables. The incentive groups indicated little, if any, potential bias. Based on the R-indicator (0.92 prepaid and 0.93 postpaid), the incentive groups had similar response deviations from the original sample. For the stratification variables, the R-indicator was nearly identical for all categories. Finally, item nonresponse was low, with less than 5% of responders missing one or more survey questions in each of the experimental groups. Finally, comparing main study respondents and follow-up study respondents showed no statistically significant differences for the two primary outcomes of interest investigated.

We compared cooperation rates, speed of response, check cashing behavior, and survey responses between the prepaid and postpaid groups using chi-square tests for testing the association between categorical variables (SUDAAN® proc. crosstab) and t-tests for continuous variables and specified categories within a categorical variable (SUDAAN® proc. descript). These analyses were performed using SAS Enterprise Guide 7.13 [18] with SAS-callable SUDAAN 11 [19]. Finally, using the differences in cooperation rates that we observed for prepaid incentives and postpaid incentives in this experiment, we estimated the potential costs of achieving our targeted number of completed surveys (*n* = 1200) using a prepaid incentive compared to a postpaid sample.

## Results

The overall survey cooperation rate was 38.0% and varied significantly by the timing of the incentive (Table 1). Cooperation rates were 41.4% in the prepaid condition and 29.1% in the postpaid condition (chi-square = 46.8, df = 1, *p*-value < 0.0001). Similar differences in cooperation rates were seen for physicians when stratified by region of the country, size of the metropolitan statistical area in which the physician was located, specialty, and gender by age. Differences in survey cooperation rates between the pre-and post-paid incentive conditions are noteworthy in the southern region of the country (41.2% in the prepaid condition compared with 25.0% in the postpaid condition) and in large metropolitan statistical areas (43.5% in the prepaid condition compared with 26.5%) in the postpaid condition). This is due to lower cooperation rates in the postpaid condition for those groups relative to postpaid cooperation rates for other regions of the country and in smaller metropolitan statistical areas. In addition, cooperation rates were highest and the differences in cooperation rate by incentive timing were lowest in the midwestern region of the country.

**Table 1:** Effect of incentive timing on physician cooperation rate, overall and by demographic characteristics

Characteristic	Cooperation rate (%) for prepaid group (*n* = 2426)	Cooperation rate (%) for postpaid group (*n* = 953)	Delta	Chi-squared statistic, degrees of freedom, *p*-value
Total	41.4 (1004)	29.1 (277)	12.3	46.8, 1, < 0.0001
Region				
Midwest	44.3 (233)	36.0 (74)	8.3	4.2, 1, 0.0406
Northeast	41.3 (240)	30.3 (70)	11.0	9.0, 1, 0.0028
South	41.2 (356)	25.0 (85)	16.2	30.6, 1, < 0.0001
West	38.7 (175)	27.5 (48)	11.2	7.5, 1, 0.0064
Metropolitan statistical area size				
Very large	41.3 (718)	29.5 (201)	11.8	30.6, 1, < 0.0001
Large	43.5 (141)	26.5 (34)	17.0	12.4, 1, 0.0005
Small/medium	40.1 (145)	29.5 (42)	10.6	5.2, 1, 0.0237
Specialty				
Hematology	39.2 (82)	26.7 (20)	12.5	4.0, 1, 0.0478
Hematology- oncology	40.5 (612)	28.3 (166)	12.2	29.2, 1, < 0.0001
Oncology	44.0 (310)	31.4 (91)	12.6	14.1, 1, 0.0002
Gender and Age				
Female	37.5 (282)	24.9 (71)	12.6	16.2, 1, 0.0001
Male under age 55	43.1 (421)	30.2 (118)	12.9	20.6, 1, < 0.0001
Male age 55 or older	43.4 (301)	32.0 (88)	11.4	11.0, 1, 0.0009

Logistic regression analysis (not shown) also demonstrated that incentive timing significantly affected the cooperation rate independently, controlling for the demographic variables, and demographic variables affect the cooperation rate controlling for incentive timing. However, the regression analysis did not identify a statistically significant interaction effect between demographic variables and incentive timing on cooperation rates.

Survey responders in the prepaid condition were also quicker to respond than those in the postpaid condition: 68.6% of responders in the prepaid condition responded before the second survey mailing, compared with only 57.4% of the responders in the postpaid condition (chi-squared = 3.4, df = 1230, *p*-value = 0.0007) (Table 2). Follow-up contacts had less effect on cooperation rates for the prepaid group than for the postpaid group.

**Table 2:** Weighted proportion of responders by contact attempt and incentive type

Incentive group	First mailing and e-mail	Second mailing and e-mail	Third mailing and prompting call	Total responders
Prepaid	68.6 (689)	14.7 (147)	16.7 (168)	100.0 (1004)
Postpaid	57.4 (159)	20.2 (56)	22.4 (62)	100.0 (277)

Check-cashing behavior also varied by timing of incentive (Table 3). In the prepaid group, 84% of responders cashed the incentive check. The prepaid condition also had a small number of incomplete responders (those who completed fewer than 85% of the survey questions that applied to them); these Incomplete responders cashed the check at a similar rate (81%). We found that non-responding physicians did not “take the money and run.” Only 6% of prepaid nonresponders cashed the check. Another 15% of ineligible sample members cashed the check.

**Table 3:** Percentage of sample members who cashed the check, by incentive condition and survey outcome

	Prepaid condition				Postpaid condition	
	Responded with a complete survey (*n* = 1004)	Responded with an incomplete survey (*n* = 47)	Did not respond (*n* = 1375)	Replied indicating they did not meet our eligibility criteria (*n* = 68)	Responded with a complete survey (*n* = 277)	Responded with an incomplete survey (*n* = 9)
Total	83.6 (839)	80.8 (38)	5.8 (80)	14.91 (10)	72.2 (200)	55.6 (5)

In the postpaid condition, 72% of survey responders cashed the check. Of the small number of incomplete responders, 56% cashed the check. Nonresponders in the postpaid condition were not given a check (Table 3).

Check-cashing behavior was similar across demographic groups (not shown); no statistically significant differences were detected.

We conducted additional analysis of survey responses by timing of incentive and found no statistically significant differences; these data are not shown.

## Discussion

The use of prepaid incentives is often recommended for improving response rates amongst physician samples [5–8]. However, few surveys of physicians have made experimental comparisons between pre- and postpaid incentive strategies that would allow researchers to quantify the potential benefits and financial risks of this approach. In this paper, we used experimental data to compare the effects of pre- and postpaid incentive checks on cooperation rates and cost.

Consistent with Delnevo et al. [11] and James et al. [12], we found cooperation rates for physicians in the prepaid condition were higher than those in the postpaid condition by 12.3 percentage points. We also found that survey responders in the prepaid condition responded earlier in the field period than those in the postpaid condition, thus requiring fewer contacts. When taken together, these findings mean that achieving the necessary number of completed surveys to power the desired substantive analyses of the survey data can be achieved through a smaller sample and fewer planned follow-up contacts when using prepaid incentives instead of postpaid incentives.

We found that demographic characteristics and incentive timing had independent effects on cooperation rates, but the effect of incentive timing on cooperation rates does not significantly differ by demographic characteristic.

This study is unique in its use of experimental data to calculate the financial implications of incentive timing. We found the financial risk of a prepaid incentive check to be low. Only 6% of nonresponding physicians in the prepaid condition cashed the check, and the numbers of ineligible or incomplete responders who cashed the check were negligible. Furthermore, a substantial portion of physicians who returned a completed survey did not cash the check.

Overall, the relatively higher cooperation rates and earlier response of the responders in the prepaid condition translated to a 30% cost savings compared with the postpaid condition. (These cost comparisons are limited to other direct costs such as printing, shipping, incentives, and labor for clerical and interviewing staff.) The amount of the cost savings will vary based on the survey’s specific design elements, including target sample, protocol for determining eligibility, survey length, incentive amount, and prompting strategy. Data for this paper are from the National Survey of Precision Medicine in Cancer Treatment, the methods for which entailed a sample of oncologists, an initial eligibility screening call, a 20-min survey, a $50 incentive provided in the form of a personalized check, two follow-up mailings, and a prompting call. See Geisen et al. [2] for additional models of cost savings associated with different hypothetical designs, including a larger starting sample and fewer follow-up contacts, for this same study.

A key feature of the prepaid incentive approach used in the National Survey of Precision Medicine in Cancer Treatment is that the prepaid incentives were provided in the form of a personalized check as opposed to cash. Cash, once provided, cannot be recovered if the respondent does not respond. However, when a check is used, researchers only incur the cost of the incentive if the check is cashed, and checks are void if not cashed within 90 days of being issued.

Another advantage of providing prepaid incentives in the form of a personalized check is that it increases the likelihood that physicians will be aware of the incentive. Survey packages may be opened by office staff and the incentive separated from the rest of the package. A personalized check made payable to the physician may be more likely to be seen by the physician [5].

## Conclusions

Investigators rely on surveys to obtain information about health care delivery from physicians that is not available from any other source. However, obtaining survey participation from physicians has become increasingly challenging. Investigators must consider the most effective use of resources to achieve sufficient physician cooperation. Our results demonstrate that when surveying physicians, the rewards of prepaid, personalized incentive checks are high, and the financial risks are low.
